# Supplementary material for: The Evolution and Characterization of the RNA Interference Pathways in Lophotrochozoa
Source: Genome Biol Evol. 2024 May 7;16(5):evae098. doi: 10.1093/gbe/evae098 (PMC11114477; doi:10.1093/gbe/evae098)
Supplement: evae098_Supplementary_Data [file evae098_supplementary_data.zip › Supplementary_materials.zip!/selected_miRNAs/NoGe_selected_miRNAs/known_miRNA_families_pdf_files/NMRB01000265.1_Notospermus_geniculatus_strain_Ushimado_scaffold265,_whole_genome_shotgun_sequence_8633.pdf]

Provisional ID	:	NMRB01000265.1_Notospermus_geniculatus_strain_Ushimado_scaffold265,_whole_genome_shotgun_sequence_8633
Score total	:	29978.1
Score for star read(s)	:	3.9
Score for read counts	:	29969.7
Score for mfe	:	1.5
Score for randfold	:	0
Score for cons. seed	:	3
Total read count	:	58796
Mature read count	:	57058
Loop read count	:	0
Star read count	:	1738

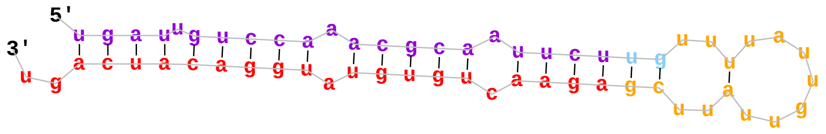

Genome coordinates: NMRB01000265.1 243296-243356

pre-miRNA sequence:
UGAUUGUCCAAACGCAAUUCUUGAUUGUCCAAACGCAAUUCUUGUUUUAUUGUUAUUCGAGAACUGUGUAUGGACAUCAGU

Mature sequence: AGAACUGUGUAUGGACAUCAGU

Aligned mature sequence of the *N. geniculatus* pre-miRNA with the top 5 hits from the BLAST search against the pre-miRNA mirGeneDB set:

```
                            ** ******* *********
Nge_Mir-219                 agaacuguguauggacaucagu
Tca-Mir-219_3p              aggacuguguguggacaucauc
Lpo-Mir-219-P22_3p          agaacuguguguggacaucagu
Lan-Mir-219_3p              agaacuguguauggacaucagu
Esc-Mir-219_3p              agaacuguguuuggacaucagu
Pmi-Mir-219_3p              agaacuguguauggacaucagu
```
